# Dental Societies

**Published:** 1860-01

**Authors:** 


### ARTICLE VII.

### *Dental Societies.*

"And as thus every action, down even to the drawing of a line or utterance of a syllable, is capable of a peculiar dignity in the manner of it, which we sometimes express by saying that it is truly done, (as a line or a tone is true,) so also is it capable of a dignity still higher in the motive of it. For there is no action so slight, nor so mean, but it may be done to a great purpose and ennobled therefore ; nor is any purpose so great but that slight actions may help it, and may be so done as to help it much * * * *.—RUSKIN.

IN continuation of some remarks we were permitted to make, on Dental Periodicals, in the last number of the Journal, we propose at present to offer a few reflections on Dental Associations, which we do not think have been so much as hinted at, much less explicitly expressed, in any of the numerous pieces on the subject with which our professional periodicals have been teeming since the late August meetings. These, in order to be the more perfectly intelligible, will be conveyed in the survey we shall take of the work done by those National Dental Societies which have from time to time, been ushered into an ephemeral existence.

We wish to know if these societies have "truly done" and for "a great purpose." If not, it is our hope that these observations may do something at least towards an avoidance in the future, of those apparent abuses to which we shall have occasion to advert. We fear indeed that here, as in the periodicals, our impartial examinations will show nothing more than very insufficient, and we had almost said, contemptible effort. From the beginning their course has been marked by failure, and as far as we know they have only been efficacious in fostering the petty spirit of competition for notoriety. Our search has been rigid through the transactions of twenty years, and during this period, we have been able to discover two circumstances which reflect credit upon them ; the first relates to the pecuniary assistance extended to a periodical which, through its previous existence and influence, had suggested the expediency of their formation ; the second, to the production of some eight or ten tolerably respectable essays which they appear to have elicited.

Here it may be objected that many eminent authorities have disputed the ability of associations to accomplish real benefit for any science, and that ours is not singular in its situation* ; we still persist, however, that societies found-

* Although anciently *philosopher* and *ascetic* were synonymous expressions—the pagans, however, discovered that "philosophy," when confined to those who secluded themselves from the world, had already become a mask for hypocrisy, as for example, with the pseudo-cynics,*—*now* it is almost universally conceded that the spirit of association is all powerful. The following testimonies bear *for* and *against*.

As *opposed* to the spirit of association, D'Israeli (*Curiosities of Literature*, "Discoveries of Secluded Men," "A Glance at the French Academy," "An English Academy of Literature") and Carlyle ("Signs of the Times,") are the only authorities that occur to us.

But as acknowledging its influence, those we shall mention and quote would appear to settle the question affirmatively. "We owe to France the justice of observing that if England had the advantage of giving birth to the discovery of *universal gravitation*, it is principally to the French geometers, and *to the encouragement held out by the Academy of Sciences*, that we owe the numerous developments of this discovery, and the revolutions which it has produced in astronomy.—*Laplace Systēm du Monde. Quoted by Brewster, Life of Newton.*

"Le principal avantage des Academies est l'esprit philosophique qui doit s'y introduire, et de la se rēpondre dans toute une nation et sur tous les ob

* See Neander, Hist. of the Church, vol. i.

ed for the advancement of dentistry, have been *particular* in their want of success. They have failed more signally. Their sole result has been to show that the right course is directly opposite to the one heretofore followed.

jets. Le savant isolé, peut se livrer sans crainte à l'esprit de systême, il n'entend que de loin le contradiction qu'il éprouve. Maisdans une société savante, le choe des opinions systematiques finit bientot pas les détruire et le dessin de se convaincre mutuellement, établit nécessairement entre les membres, la convention de n'admettreque les resultats de l'observation et du calcul."—*Laplace, quoted by Weld.*

"The development and advancement of science are *signally* indebted to those associations: the Academy del Cimento at Florence, which endured but for a short time; the Royal Society of London, and the Academy of Sciences at Paris." *Weld's Hist. of the Royal Society.*

"The literary institution * * * * * that has been most successful in promoting the interests of science, is that of the Royal Academy of Science of Paris, *where small pensions and great honors* * * * * * have been the means of advancing the mathematical sciences to a state of unexampled prosperity. In England *where such an institution as that just mentioned was wanting*, where the public is perpetually prepared with the question *cui bono*, the same progress has not been made."—*Prof. Playfair. Quoted by Brewster.*

"The spirit of association * * * * * is of native and not of recent growth in England; and among the modern monuments and public works of London, or indeed in the British empire at large, there is scarcely one that is striking or of any magnitude but what has its root in this principle. The British empire in India, the greatest in the world, has grown up in less than a century from a company of traders and capitalists."—*Chevalier Bunsen. Signs of the Times.*

"The only mode of supplying with any certainty this want"—(the point of sight from which the totality of science may be observed and directed)—"is to be sought in the combination of men of science, representing all the specialties and working together for the common object of preserving that unity and presiding over that general direction. This has been to some extent done in many countries by the establishment of academies embracing the whole range of the sciences * * * * * In the absence of such an institution in this country, all lovers of sciences must rejoice at the existence and activity of this Association, which embraces in its sphere of action, if not the whole range of sciences, yet a very large and important section of them.—*Prince Albert's speech before the British Association,* 1859.

"The discovery of new facts in physical science is less effectually promoted by genius than by *co-operation.*"—*Sir W. Hamilton. Epistolæ Obscurorum Virorum.*

"The great modern revival of classical learning resulted in a great degree from the community of ideas—*co-operation* of the scholars of St. Agnes," (A convent in Westphalia.)—*Sir W. Hamilton. Idem.*

This announcement will astonish many, yet we do not make it upon individual authority; we have the "proceedings" before us, which we will now proceed to examine.

"The American Society of Dental Surgeons" met for the first time in New York on the 18th day of August, 1840. The most intelligent dentists in America were either personally present or represented. Some misgivings were felt by many as to the feasibility of the project, yet, on the whole, much unanimity of sentiment prevailed. A constitution, which redounds to the honor of its framers, was offered and adopted. It, paradoxically, but sanguinely announced that the object of the society was the advancement of science by *free communication* among its members and, with such an elevated aim, however encumbered by the condition of "free communication" which practically means "evil communication," the society appeared to be destined to succeed. No less than twelve essays, calling for a vast amount of professional erudition, were allotted to different members. Enthusiasm carried the day. Every one was anxious to work, and so they adjourned. As we shall see this programme, of organizing, appointing work enthusiastically, and adjourning, became the routine of all the societies.

During the following year the interest was kept up by constant notices in the Journal, and when the society met in Philadelphia, no abatement in the general feeling of enthusiasm was discernible; but out of the twelve essays, the subjects of which had been publicly rehearsed at the preceding meeting and printed in large type, not one was read. In their place one was offered on a subject which

Sir Humphrey Davy spoke of the Royal Society as the "*elder brother of Science.*"—*Speech on the Progress and Objects of Science.*

While Carlyle's "Signs of the Times" on the whole, is a vehement denunciation of the spirit of *Mechanism,* (or Association,) it is full of satirical, but forced acknowledgments of its pervading power. These are too numerous, throughout the essay, to permit of more than bare reference, particularly as our note has already reached unwieldy limits.

had not been mentioned but was nevertheless of interest and is at present valuable. There was also an "Opening Address," and a "Dissertation" of two pages on things in general. Ten gentlemen had the satisfaction of receiving appointments to read "dissertations" at the next annual meeting, and the society adjourned. In Boston there was a large attendance. Much general activity was displayed and the list of membership increased ; but instead of ten dissertations, science was obliged to put up with the inevitable "Opening Address," *three* essays, relating to the real objects of the society, and an ethical production by the muse of the profession.

At this epoch in our history personal difficulties began to appear in the place of "advancement of science ;" but as none of these have yet assumed the importance of Leibnitzio-Newtonian controversy we shall not attempt to elucidate them.

Seven dissertations were appointed for the next year and of that number, besides the address, three or four only were presented. The dismal farce of announcing names and subjects was, as usual, gone through with. The gradual diminution in the number appointed and read continued. Five were allotted for the coming year. One, on salivary calculus was read.

In the meantime local societies were starting into being. As might have been anticipated, they were prolific of opening and closing speeches, of hints oft' told, of quackery, of constitution, amendments and the like ; but of true scientific fruit they produced nothing. The parent tree bore all that was to be gathered.

The "American Society" had by this time reached, what we may analogously call, its full vigor and now began rapidly to decline. Although the radical defects of its organization at this period appear in all their deformity it is refreshing to notice that a few devoted men, out of a hundred or so of members, still preserved the best interests of their profession at heart, and did all that was done in the way of study. Their contributions were treated as of little importance, however, as matters of a personal, and hence of a more interesting character, consumed the hours of meeting. The pressure from without became too great and the almost criminally weak barriers as to the qualifications necessary to membership gave way. Persons were indiscriminately admitted and the usual disturbances ensued. Many of those who still preserved their self-respect withdrew, and the society, now unsustained, tottered through two or three years of puerility and died.

Out of the ruins sprang that miracle of republicanism, the American Dental Convention. In pursuance of a call, a large number of dentists met in Philadelphia on the 2nd of August, 1855. Eighty persons signed the hastily prepared articles of association, and as these articles are a wonder in their way, we beg to quote the more striking parts:

"*Preamble.*—At a national convention of dentists assembled in Philadelphia on the 2nd day of August, 1855, for the purpose of consulting upon the measures best adapted *to advance the cause of science*, and *the common interest* and *honor** of the profession, the following plan of organization was adopted:—

"Art. II. The association is intended to promote professional and personal intercourse among those who are engaged in the cultivation and practice of dentistry throughout the world; *to advance the cause of dental education*, and *systematize* and *strengthen* the *exertions of its friends*, and, by a mutual interchange of opinions and experience to advance the knowledge and liberalize its relations."

"Art. III. The convention shall consist of the members of the convention who shall sign these articles of association, and of such other practitioners of dentistry, and

* The italics are our own.

*auxiliary branches* of *science** as shall hereafter be elected to membership, and in like manner sign these articles."

What we have quoted contains all that is characteristic in the organization of the convention, and, without the further proof contained in the "proceedings" of every successive year, we think that the practical defeat of the high sounding intentions of the preamble is discernible in the absurdly unwise omission of all reference to necessary qualifications.

That the members of a profession pretending to have some elements of the liberal in it, should solemnly meet together, "pursuant to a call," and enact such laws, by which the most limpid intellect might have foreseen their inevitable destruction by all manner of idle talkers and enterprising speculators, is indeed surprising; and the consequences were not slow in appearing. It must be admitted, however, that the great evil of easy admission was not created by dental societies; other societies have suffered from the same cause, and many of them, including the American Association for the Advancement of Science, have endeavored to abate it by creating two classes of members and by other enactments. But to return—

The first convention, having come into an unhealthy life by a sort of "feet presentation," immediately exhibited remarkable signs of vitality by appointing committees and discussing that mighty subject of filling teeth. A resolution was also passed which recommended the formation of local societies; this profoundedly unoriginal act was the best of the session, but the question as to the expediency of sponge gold, absorbed the almost undivided attention of the learned body. A question which should have been determined by every one at home, by a few weeks experiment, was brought up and kept up to the exclusion of as yet unsettled but deeply important and interesting physi-

*Those "auxiliary branches of science" are instrument-making, tooth-manufacturing, and material-preparing in general.

ological considerations. Gentlemen gave their "experience," according to article 11, as to the application of a dossel of cotton and the drawing of a file. They made "brief," but it is to be hoped for the intelligence of the profession, unsatisfactory, "remarks" about plaster. They thought well of india rubber, and not unfrequently the ever blooming, perennial theme, alveolar abscess, engaged their valuable thoughts.

Now we do not wish to insinuate that dentists are to be ashamed of gold, for it is by gold that they live ; nor would we seek to impugn the valuable properties of cotton ; the file is indispensable in the operating room as well as in the laboratory, and plaster is unavoidable. But admitting the value and even dignity of all these things, why should unceasing changes be rung upon their mere names ? New properties are not evolved by crying "gold, gold," or "cotton, cotton," on the contrary, we discover a fact which was, maybe, never unknown, namely, that the crier is wanting in a certain property—common sense. Voltaire was very true when he said, "Le sens commun n'est pas si commun." No occult principle has yet been signalized by which a man can make a file more or less than a file by repeating its appelative. Something more must be done if we would add to the truths of science or even present the old ones more clearly. Instead of superficial, one sided experience —which is worse than worthless, as it is pernicious—our meetings should indicate patient study and careful observation. Agassiz says that "though at times a subject may seem to have lost some of its value for being less novel, it generally gains tenfold in scientific importance by being presented in the fullest light of all its natural relations."* The application of this remark has particular reference to the isolated, barren facts which are frequently thrown out at the dentists' meetings.

* Contributions to the Nat. Hist. of the U. S. Preface, vol. i.

An intelligent person out of the profession, if he thought at all on the subject, would suppose that dentists meet above all to exchange ideas relating to the subjects of their respective studies during the past year; that intelligent person, however, would be immensely mistaken, they meet to re-talk what they talked the year before. Their united wisdom is employed in appointing committees, not in attempting to settle a difficult question. They practice in the parliamentary exercise of passing resolutions, in organizing and in disbanding. They visit various cities and draw down the ridicule of the press by their dismal efforts at pleasantries touching their capacity as eaters.* Let us, however, give all due praise to the discussions of the second convention; here we had decidedly less idle talk than at any meeting of dentists ever held in America. The members entered, with a degree of seriousness, into the study of an interesting question and the meeting was on the whole quite creditable. Several persons presumed to talk about that which they did not adequately comprehend and in place of appropriate and intelligent surmise they threw out experience not at all in point. Still, with these trifling exceptions, the hours of the convention were well spent.

The meeting of 1857 was a feeble imitation of that of the preceding year. Several hints were dropped which might have been made valuable, but the proceedings were, as a whole, lamentably invaluable.

The fourth meeting was without any feature of interest whatever, and the fifth is chiefly noticeable for the good opinion some of the members appeared to entertain of it. One, in the exuberance of his admiration, spoke of the convention as second to none in the world, which was a very high compliment indeed; and the President in his opening remarks, eulogized it for its courtesy and excellent deportment—two features which we had hitherto assumed to be always present when gentlemen meet. He might

* Witness the transactions at Boston.

have commended them with the same propriety, for their personal cleanliness, or for having their hair brushed. He also, with singular infelicity, but doubtless most good naturedly, alluded to the (vinous?) sobriety of the convention and its freedom from other excess. In commenting upon these compliments, the most unpleasant questions might be asked and the answer would be, that, polite and sober as some might be willing to confess, the convocations of the dental convention are never disturbed by annoying inquiries into abstruse questions of science. So much for the convention ; but before dismissing it from our consideration we have a few interrogatories to put, which have direct reference to the implicit and explicit promises of the articles of association :—

Has the convention taken one step to advance dental education?

Has it done anything towards "systematizing and strengthening the exertions of its friends?"

Has it taken any step by which the cause of science may expect to be advanced?

Has the convention maintained the honor of the profession? To all of these there is but one reply—no.

The two dental societies of London, notwithstanding their affirmed instability, claim greater consideration than, for our present purpose, we can bestow. We may, nevertheless, observe that they have assumed a more dignified attitude than could have been expected, from the great social prejudices which are known to exist around them. Without, as yet, having contributed anything of value to science, their transactions are by no means worthless. The volume published by the Odontological Society is handsomely printed and expensively illustrated. Some of the papers are well written, promising much for the future.

The College of Dentists, if we may speak of it as a "society," in our sense, deserves commendation also, for the interesting lectures which we have been able to peruse through its instrumentality.

Thus, after frankly confessing the utter inefficiency of our two great societies, one naturally asks : Does our want of success result from the inherent deficiencies of dentistry or from the faults of organization ? We respond that the difficulty is not in the meagreness of dentistry but in unwise organization. The primary objection has been excessive freedom of access, which, of itself, is sufficient to entail all kinds of trouble and eventual dissolution. Most of the learned societies of Europe have taken great precautions in the admission of members, and we strongly insist, that some measures of the same kind are necessary in dental societies. To be sure, they are not likely to rival those European institutions in distinction, yet, they as imperatively demand similar barriers to the incursions of the "great unwashed." Notwithstanding the flattering remarks of members of the convention, it cannot be taken for granted that all dentists, or indeed all members of any profession, are prepared for admission into every assemblage.

Having assumed that this evil of freedom of access needs only to be stated to be duly appreciated—having the history of societies before us—the next point is, how shall the evil be avoided ? It would be ridiculous to apply the test of scientific reputation or proficiency, and we have only the tests of morals and money left for our present needs. The importance of manners and morals will no doubt receive due consideration, as heretofore, in future "opening addresses," we will, therefore, proceed to the more tangible subject ; but without going articulately into a discussion of the details of a model organization, it is certain that our chief evil may be greatly mitigated by the money test. There can be no doubt that considerable fees will keep out a large class of persons who are not of the least use and who, under the present order of things, are exceedingly troublesome. There is another class, unfortunately, as much to be dreaded ; it consists of those who are willing to make large outlays in the gratification of vanity, who *must* be known as participants in every movement. The five or ten minutes rule is the only check we can think of to be applied here. They penetrate everywhere, and if we cannot absolutely prevent we can at least use palliatives. Besides keeping out the troublesome, the treasury will be filled and this is indispensable, under any circumstances, for the accomplishment of the objects of association.

The following table will indicate the custom of the principal British societies in regard to fees. Most, if not all, of these are metropolitan and possess fixed places of meeting, together with libraries and museums ; still the sums which they spend in the acquisition of this property should be used, by a national association of dentists, for purposes equally, if not more, desirable. It should bestow the money it would spend, if fixed, in a library and museum, in prizes and in the support of scientific investigation. The value of our table, therefore, is not impaired by the difference of circumstance between the societies we shall refer to and a dental association on new principles. Money is as necessary in perambulatory as in fixed societies.*

| | | Admission Fee. | Per Annum. |
|---|---|---|---|
| The Royal Society, | | $50 00 | $20 00 |
| The Geological Society, | non-residents, | 50 00 | |
| | residents, | 30 00 | 15 00 |
| The Linnæan Society, | | 30 00 | 15 00 |
| The Royal Med. and Chirurgical Society, | | 30 00 | 15 00 |
| The Zoölogical Society, | | 25 00 | 15 00 |
| The Royal Society of Literature, | | 15 00 | 10 00 |
| The Royal Astronomical Society, | | 10 00 | 10 00 |

The practice of these eminent bodies, in connection with the testimony of our own unhappy experience, is such that we consider the propriety of large fees as affirmatively settled. Except under peculiar circumstances, as, for in-

*The British Association for the Advancement of Science, a perambulatory society, has spent in round numbers, $85,000, (£17,000,) in the accomplishment of scientific objects.

stance, when there are grades of membership, a grade for work and two or three grades of members who facilitate operations by supplying funds, we cannot conceive of any advantage accruing from small fees. Genuine lovers of truth, philosophers, will rarely withhold their co-operation on account of an increased tax. Their devotion is not great in proportion to the smallness of the costs. At first view it may be supposed that poor men suffer by this arrangement, for the good of the majority; but, upon reflection, it will appear that they will be benefited, their service being always rewarded if of value.

Supposing then that a large fee has been determined upon, what shall the dentists do in conclave assembled? Our idea is that their workings should be modeled, with certain modifications, after those great representative societies we have alluded to. A careful study of their history and rules will enable us to anticipate most of the difficulties to be apprehended, and with this basis of knowledge for our operations, we may then add such distinctive features as the profession requires.

The utmost liberality should be displayed in giving prizes. It must be remembered that many, who would devote themselves to scientific pursuits, even for a limited period in the investigation of suddenly suggested subjects, are deterred by pecuniary difficulties. Books, apparatus, materials of every description are expensive, and without a prospective return in some shape, they must of necessity keep at their "bread and butter science." Moreover, prizes stimulate those who would otherwise make no exertion. But it is useless to recapitulate the advantages of prizes. The example of the greatest institutions all over the world must be familiar to all.*

*The following particulars, relating to the prizes in the gift of the Royal Society, are illustrative of our subject; possibly they may be of use:

In 1736 Sir Godfrey Copley's donation was converted into a gold medal of the value of five pounds, bearing the arms of the society, as Weld the historiographer of the society remarks: "This medal, the most honorable in the

The student then should be encouraged by emoluments and honors, to re-examine old truths with greater attention and to follow out new speculations undismayed by possible loss. Physiology, microscopical and comparative anatopower of the society to bestow, (termed by Sir Humphrey Davy the ancient olive-crown of the Royal Society,) has been awarded for upwards of a hundred years to the authors of brilliant discoveries; and there is hardly a name eminent in science that does not appear as the recipient of this honorable testimonial of appreciated merit."

In 1796 Count Rumford gave five thousand dollars, the interest of which was to be given in the shape of two medals—one of gold, the other of silver—"as a premium to the author of the most important discovery or useful improvement on Heat, or on Light."

In 1825 the king founded two gold medals of the value of two hundred and fifty dollars each, which are now bestowed under the following conditions: That the royal medals be given for such papers *only* as have been presented to the Royal Society, and inserted in their transactions.

That the triennial cycle of subjects be:

1. Astronomy; *Physiology.*
2. Physics; Geology, or Mineralogy.
3. Mathematics; *Chemistry.*

In 1825 Dr. Wallaston established the *Donation Fund* by giving $10,000 to the society, the dividends of which are applied to the promotion of experimental researches, &c., in rewarding those by whom such may have been made." Mr. Gilbert contributed $5,000 in addition to this fund and Mr. Hatchett $500. In 1848 the fund was raised to the sum of $25,000.

In 1839 the celebrated Bridgewater gift of $40,000 was made for purposes which are so well known that they need not now be enumerated. Subsequent gifts have doubtless been made but, if ever aware of them, we cannot now recall them to mind.

Since writing the above the grants of the British Association, for 1859, have been announced in *the Athenæum:* the following is a list of the investigators chosen, the topics they are to treat, and the sums of money they are to receive towards the expenses:—to the Kew Observatory, 500*l.*,—to Prof. Sulli van, "Solubility of Salts," 30*l.*,— to Prof. Voelcker, "Constituents of Manures," 25*l.*,—to Mr. A. Gage, "Chemico-Mechanical Analysis of Rocks," 25*l.*,—to Dr. A. Smith, "Scientific Evidences in Courts of Law," 10*l.*,—to R. Mallet, "Earthquake Waves," 25*l.*,—to Rev. Dr. Anderson, "Excavations in Yellow Sandstone of Dura-Den," 20*l.*,—to Sir R. J. Murchison, "Fossils in Upper Silurian Rocks Lesmahoge," 15*l.*,—to R. M. Andrews, "General Dredging," 50*l.*,—to Dr. Ogilvie, "Dredging North and East Coasts of Scotland," 25*l.*,—to Prof. Kinahan, "Dredging in Dublin Bay," 15*l.*,—to Dr. Daubeny, "Growth of Plants," 10*l.*,—to Prof. Allman, "Report on Hydroid Zoophytes," 10*l.*,—to Dr. Wilson, "Colour Blindness," 10*l.*,—to Admiral Moorsom, "Steam-Vessels Performance," 150*l.*,—and to Prof. J. Thompson, "Discharge of Water," 10*l.*;—making altogether a total of 930*l*.

my, chemistry and pathology, in reference to dental science and operative and mechanical dentistry, should be standing subjects for prize theses.* Most valuable results would inevitably follow.

Care should be taken, above all, to avoid that common error of magnifying the importance of mere handicraft dexterity. For,as those branches of professional knowledge are pursued which require the higher faculties of the mind for their development, so will dentistry be entitled to a place among the liberal professions. The observance of this truth, the violation of which has heretofore justly caused the dentist to be looked upon as a mere craftsman, deeply concerns all future advancement. It should be ever borne in mind.

From the foregoing it is plain that our reform consists, *first*, in so constituting any national society of dentists that, if possible, only the meritorious be admitted. This may be done by requiring every candidate to be recommended by at least three members and the votes of two-thirds of those present as necessary to election ; furthermore, an admission fee of the largest reasonable amount—say of from fifteen to twenty-five dollars—and a proportionably large yearly payment.

*Secondly*, we would accept the teachings of universal experience and stimulate industry to the utmost by founding prizes for discoveries, inventions, and thesis. And the invitation to competition should not be confined to the members of the society or the profession. But these prizes should not be conferred lightly. Unquestionable merit,

*It has occurred to us that dentists might apply themselves, with the greatest profit, to the *morphology of the teeth*. It is possible, as the teeth have so marked a relation to the necessities of the animal, that their more hidden peculiarities, particularly in the human species, may indicate most interesting psychological modifications. This has been suggested by some remarks made by Agassiz, upon the importance of studying the habits of animals, (contributions, p. 57—63, vol. 1,) and by the valuable results of the comparatively recent sciences *vegetable and animal morphology*.

to be determined by rigid examination, alone should entitle to honor.

And *thirdly*, as we conceive that dentistry can only advance as it cultivates science, the highest honors should be bestowed on those branches of study which are scientifically important but only very *indirectly* valuable in *practice;* these are Physiology, Microscopical and Comparative Anatomy, Chemistry, etc.

Thus the real wants of the profession would be measurably supplied.

