# Erratum

**Published:** 1860-01

**Authors:** 


*Erratum.*—The author of the article on dental societies has requested us to insert the words "*in Germany*" after the word "learning," in the second note from Hamilton.

